# (1*S*,2*S*,6*R*,7a*R*)-2-Benzyl-1,6-dihy­droxy­hexa­hydro­pyrrolizin-3-one

**DOI:** 10.1107/S1600536812002334

**Published:** 2012-02-04

**Authors:** F. L. Oliveira, K. R. L. Freire, R. Aparicio, F. Coelho

**Affiliations:** a Laboratory of Structural Biology and Crystallography, Institute of Chemistry, University of Campinas, CP6154, CEP 13083-970, Campinas-SP, Brazil; b Laboratory of Synthesis of Natural Products and Drugs, Institute of Chemistry, University of Campinas, CP6154, CEP 13083-970, Campinas-SP, Brazil

## Abstract

In the title compound, C_14_H_17_NO_3_, the dihedral angles show that the H atoms at two stereocenters are in a *trans*-diaxial configuration. In the crystal, the molecules are linked by O—H⋯O hydrogen bonds. The absolute configuration of the molecule has been established on the basis of refinement of the Hooft and Flack parameters.

## Related literature

For a synthetic sequence for the preparation of the title compound, see: de Luna Freire *et al.* (2011 ▶). For the use of this type of compounds as LFA-1 (Lymphocyte Function-Associated Anti­gen-1) inhibitors, see: Baumann (2007 ▶). For a related structure, see: Newton *et al.* (2004 ▶).
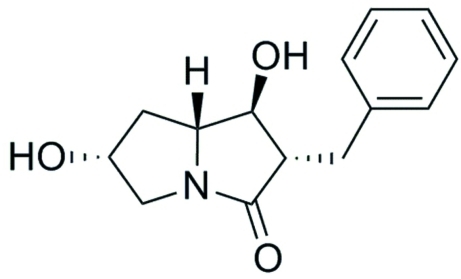


## Experimental

### 

#### Crystal data

C_14_H_17_NO_3_
*M* *_r_* = 247.29
Orthorhombic, 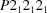

*a* = 6.6241 (3) Å
*b* = 13.6873 (6) Å
*c* = 13.9726 (6) Å
*V* = 1266.84 (10) Å^3^
*Z* = 4
Cu *K*α radiation
μ = 0.74 mm^−1^
*T* = 100 K
0.17 × 0.15 × 0.12 mm

#### Data collection

Bruker Kappa APEXII DUO diffractometer
26923 measured reflections
2295 independent reflections
2290 reflections with *I* > 2σ(*I*)
*R*_int_ = 0.026

#### Refinement

*R*[*F* ^2^ > 2σ(*F* ^2^)] = 0.026
*wR*(*F* ^2^) = 0.078
*S* = 1.15
2295 reflections
172 parameters
H atoms treated by a mixture of independent and constrained refinement
Δρ_max_ = 0.17 e Å^−3^
Δρ_min_ = −0.16 e Å^−3^
Absolute structure: Flack (1983 ▶) and Hooft *et al.* (2008 ▶) [Hooft parameter = 0.00(2), (943 Bijvoet pairs)]
Flack parameter: 0.00 (16)

### 

Data collection: *APEX2* (Bruker, 2010 ▶); cell refinement: *SAINT* (Bruker, 2010 ▶); data reduction: *SAINT*; program(s) used to solve structure: *SHELXS97* (Sheldrick, 2008 ▶); program(s) used to refine structure: *SHELXL97* (Sheldrick, 2008 ▶); molecular graphics: *WinGX* (Farrugia, 1999 ▶) and *PLATON* (Spek, 2009 ▶); software used to prepare material for publication: *publCIF* (Westrip, 2010) ▶ and *PLATON*.

## Supplementary Material

Crystal structure: contains datablock(s) I, global. DOI: 10.1107/S1600536812002334/pv2494sup1.cif

Structure factors: contains datablock(s) I. DOI: 10.1107/S1600536812002334/pv2494Isup2.hkl

Supplementary material file. DOI: 10.1107/S1600536812002334/pv2494Isup3.cml

Additional supplementary materials:  crystallographic information; 3D view; checkCIF report

## Figures and Tables

**Table 1:** Hydrogen-bond geometry (Å, °)

*D*—H⋯*A*	*D*—H	H⋯*A*	*D*⋯*A*	*D*—H⋯*A*
O3—H3*A*⋯O2^i^	0.93 (2)	1.73 (2)	2.6395 (12)	164 (2)
O1—H1*A*⋯O3^ii^	0.86 (2)	1.93 (2)	2.7716 (13)	167 (19)

Symmetry codes: (i) 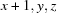; (ii) 
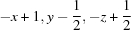
.

**Fig. 1.**
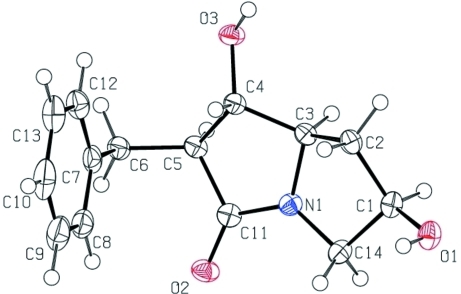
Molecular view of the title compound showing the atom-labeling scheme. Displacement ellipsoids are drawn at the 50% probability level. H atoms are presented as a small spheres of arbitrary radius.

**Fig. 2.**
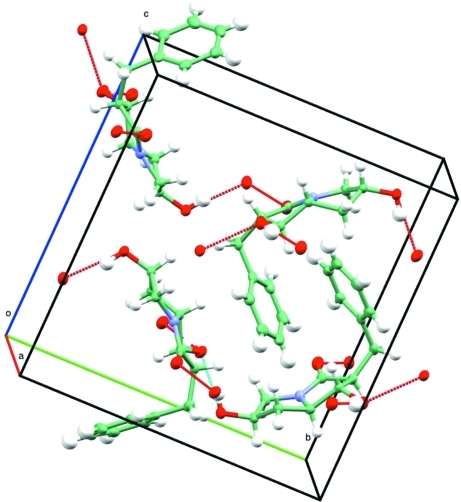
A unit cell packing diagram of the title compound showing hydrogen bonds as dashed lines.

## supplementary crystallographic information

### Comment

The title compound can be used as a prototype for the development of new inhibitors of LFA-1 (lymphocyte function-associated antigen 1) with potential application as anti-inflammatory agents (Baumann, 2007). The title compound is a new asymmetric benzyl-pyrrolizidinone which has been synthesized in our laboratory and its crystal structure is presented in this article.

The title compound (Fig. 1) has four stereocenters and was prepared from a Morita-Baylis-Hillman adduct. The dihedral angles H3—C3—C4—H4 = -158° and H4—C4—C5—H5 = 163° show that H atoms 3, 4 and 5 at the two new stereocenters are in a *trans*-diaxial configuration. These values agree with the coupling constant values obtained for these H atoms in the ^1^H NMR analysis. The crystal structure is stabilized by intermolecular hydrogen bonds (Tab. 1 & Fig. 2).

### Experimental

The title compound was prepared using a synthetic sequence described in the literature (de Luna Freire *et al.*, 2011) and purified by flash silica gel column chromatography (CH_2_Cl_2_:MeOH – solvent gradient: 0:100 to 97:03) to afford 0.06 g (as a white solid) in 97% yield. It was then recrystallized using the liquid-vapor saturation method, dissolved in ethanol and crystallized with a vapor pressure of a second less polar liquid (ethyl ether), in a closed camera, providing the slow formation of crystals.

### Refinement

The H-atoms bonded to C-atoms were included in the refinements at geometrically idealized positions with C—H = 0.95, 0.99 and 1.00 Å, for aryl, methylene and methyne H-atoms, respectively, with and *U*_iso_(H) = 1.2 times *U*_eq_(C). The H-atoms bonded to O atoms were allowed to refine freely. The Flack parameter was *x*=0.00 (16) (Flack, 1983). Further analysis of the absolute structure was performed using likelihood methods (Hooft *et al.*, 2008) with *PLATON* (Spek, 2009). A total of 943 Bijvoet pairs were included in the calculations. The resulting value of the Hooft parameter was *y* = 0.00 (2), with a probability for an inverted structure smaller than 1x10^-100^. These results indicated that the absolute structure has been correctly assigned.

### Crystal data

**Table:**

C_14_H_17_NO_3_	*D*_x_ = 1.297 Mg m^−^^3^
*M**_r_* = 247.29	Cu *K*α radiation, λ = 1.54178 Å
Orthorhombic, *P*2_1_2_1_2_1_	Cell parameters from 2295 reflections
*a* = 6.6241 (3) Å	θ = 4.5–69.5°
*b* = 13.6873 (6) Å	µ = 0.74 mm^−^^1^
*c* = 13.9726 (6) Å	*T* = 100 K
*V* = 1266.84 (10) Å^3^	Rectangular, colourless
*Z* = 4	0.17 × 0.15 × 0.12 mm
*F*(000) = 528	

### Data collection

**Table:**

Bruker Kappa APEXII DUO diffractometer	2290 reflections with *I* > 2σ(*I*)
Radiation source: fine-focus sealed tube	*R*_int_ = 0.026
Graphite monochromator	θ_max_ = 69.5°, θ_min_ = 4.5°
Bruker APEX CCD area–detector scans	*h* = −7→7
26923 measured reflections	*k* = −15→16
2295 independent reflections	*l* = −16→16

### Refinement

**Table:**

Refinement on *F*^2^	Hydrogen site location: inferred from neighbouring sites
Least-squares matrix: full	H atoms treated by a mixture of independent and constrained refinement
*R*[*F*^2^ > 2σ(*F*^2^)] = 0.026	*w* = 1/[σ^2^(*F*_o_^2^) + (0.0491*P*)^2^ + 0.1808*P*] where *P* = (*F*_o_^2^ + 2*F*_c_^2^)/3
*wR*(*F*^2^) = 0.078	(Δ/σ)_max_ = 0.001
*S* = 1.15	Δρ_max_ = 0.17 e Å^−^^3^
2295 reflections	Δρ_min_ = −0.16 e Å^−^^3^
172 parameters	Extinction correction: *SHELXL97* (Sheldrick, 2008), Fc^*^=kFc[1+0.001xFc^2^λ^3^/sin(2θ)]^-1/4^
0 restraints	Extinction coefficient: 0.0086 (8)
Primary atom site location: structure-invariant direct methods	Absolute structure: Flack (1983) and Hooft *et al.* (2008) [Hooft parameter = 0.00(2), (943 Bijvoet pairs)]'
Secondary atom site location: difference Fourier map	Flack parameter: 0.00 (16)

### Special details

**Table:**

Experimental. [α]_D_^20^ + 51 (c 1, MeOH); *M*. p. 135–136° C; IR (KBr, *v*_max_): 3404, 3232, 2987, 2936, 2897, 2871, 1670, 1447, 1416, 1375, 1300, 1263, 1222,1175, 1121 cm-1; 1H NMR (500 MHz, CD_3_OD) δ 1.55 (dddd, J = 13.4, 5.3, 4.0, 1.0 Hz, 1H, H-2 A); 2.25 (ddd, J = 13.4, 8.0, 5.4 Hz, 1H, H-2B); 2.93 (m, 2H, H-8, H-5); 3.02 (m, J = 7.5, 1.8 Hz, 1H, H-6); 3.08 (ddd, J = 12.0, 4.9, 1.3 Hz, 1H, H-14 A); 3.52 (dd, J = 12.0, 2.4 Hz, 1H, H-14B); 3.64 (m, J_H3,H4_ = 7.0, J = 8.0, 5.3 Hz, 1H, H-3); 3.88 (dd,J_H4,H5_ = 9.4, J_H3,H4_ = 7.0 Hz, 1H, H-4); 4.41 (m, J = 5.1, 4.0, 3.0 Hz, 1H, H-1); 7.15 (m, 1H, H—Ar); 7.23 (m, 2H, H—Ar); 7.29 (m, 2H, H—Ar); ^13^C NMR (62.5 MHz, (CD_3_)_2_CO) δ 34.4, 38.6, 52.3, 54.0, 65.6, 72.4, 80.6, 126.5, 128.7, 130.3, 141.0, 175.6; HRMS (ESI-TOF) Calcd. for C_14_H_18_NO_3_ [*M* + H]^+^ 248.1287. Found 248.1286.
Geometry. All e.s.d.'s (except the e.s.d. in the dihedral angle between two l.s. planes) are estimated using the full covariance matrix. The cell e.s.d.'s are taken into account individually in the estimation of e.s.d.'s in distances, angles and torsion angles; correlations between e.s.d.'s in cell parameters are only used when they are defined by crystal symmetry. An approximate (isotropic) treatment of cell e.s.d.'s is used for estimating e.s.d.'s involving l.s. planes.
Refinement. Refinement of *F*^2^ against ALL reflections. The weighted *R*-factor *wR* and goodness of fit *S* are based on *F*^2^, conventional *R*-factors *R* are based on *F*, with *F* set to zero for negative *F*^2^. The threshold expression of *F*^2^ > σ(*F*^2^) is used only for calculating *R*-factors(gt) *etc*. and is not relevant to the choice of reflections for refinement. *R*-factors based on *F*^2^ are statistically about twice as large as those based on *F*, and *R*- factors based on ALL data will be even larger.

### Fractional atomic coordinates and isotropic or equivalent isotropic displacement parameters (Å^2^)

**Table:**

	*x*	*y*	*z*	*U*_iso_*/*U*_eq_	
O1	0.38278 (15)	0.66691 (6)	0.06882 (7)	0.0253 (2)	
O2	−0.07013 (13)	0.91601 (7)	0.27608 (7)	0.0264 (2)	
O3	0.61142 (13)	1.03167 (6)	0.28357 (7)	0.0238 (2)	
N1	0.21491 (16)	0.88149 (7)	0.19167 (7)	0.0204 (3)	
C1	0.3664 (2)	0.76715 (9)	0.09195 (9)	0.0217 (3)	
H1	0.3789	0.8046	0.0308	0.026*	
C2	0.52450 (19)	0.80862 (9)	0.16169 (9)	0.0216 (3)	
H2A	0.5399	0.7667	0.2189	0.026*	
H2B	0.6575	0.8168	0.1304	0.026*	
C3	0.42952 (18)	0.90724 (9)	0.18696 (9)	0.0193 (3)	
H3	0.4529	0.9549	0.1338	0.023*	
C4	0.46008 (18)	0.95809 (9)	0.28423 (9)	0.0192 (3)	
H4	0.4913	0.9084	0.3346	0.023*	
C5	0.25242 (19)	1.00357 (9)	0.30318 (9)	0.0200 (3)	
H5	0.2442	1.0650	0.2646	0.024*	
C6	0.20002 (19)	1.03016 (9)	0.40713 (9)	0.0235 (3)	
H6A	0.2759	1.0898	0.4249	0.028*	
H6B	0.0544	1.0461	0.4105	0.028*	
C7	0.2453 (2)	0.95143 (9)	0.48012 (9)	0.0256 (3)	
C8	0.1026 (3)	0.87967 (10)	0.50128 (10)	0.0335 (3)	
H8	−0.0244	0.8801	0.4697	0.040*	
C9	0.1451 (3)	0.80737 (11)	0.56844 (10)	0.0428 (4)	
H9	0.0466	0.7591	0.5826	0.051*	
C10	0.3288 (3)	0.80545 (11)	0.61435 (10)	0.0421 (4)	
H10	0.3573	0.7556	0.6597	0.050*	
C11	0.10960 (19)	0.92968 (8)	0.25733 (9)	0.0210 (3)	
C12	0.4306 (2)	0.94914 (10)	0.52735 (9)	0.0288 (3)	
H12	0.5291	0.9976	0.5137	0.035*	
C13	0.4730 (3)	0.87670 (11)	0.59435 (10)	0.0372 (4)	
H13	0.5996	0.8759	0.6263	0.045*	
C14	0.1616 (2)	0.79469 (9)	0.13686 (9)	0.0235 (3)	
H14A	0.0595	0.8096	0.0872	0.028*	
H14B	0.1101	0.7420	0.1788	0.028*	
H3A	0.737 (4)	1.0009 (16)	0.2841 (14)	0.050 (5)*	
H1A	0.379 (3)	0.6326 (15)	0.1204 (16)	0.043 (5)*	

### Atomic displacement parameters (Å^2^)

**Table:**

	*U*^11^	*U*^22^	*U*^33^	*U*^12^	*U*^13^	*U*^23^
O1	0.0350 (5)	0.0173 (4)	0.0237 (4)	0.0039 (4)	0.0009 (4)	−0.0011 (3)
O2	0.0173 (4)	0.0252 (4)	0.0368 (5)	0.0013 (4)	0.0018 (4)	−0.0020 (4)
O3	0.0171 (4)	0.0209 (4)	0.0334 (5)	−0.0010 (4)	0.0019 (4)	−0.0044 (4)
N1	0.0183 (5)	0.0200 (5)	0.0229 (5)	0.0017 (4)	−0.0018 (4)	−0.0007 (4)
C1	0.0283 (7)	0.0173 (6)	0.0194 (6)	0.0033 (5)	−0.0009 (5)	0.0005 (5)
C2	0.0212 (6)	0.0210 (6)	0.0226 (6)	0.0038 (5)	0.0022 (5)	−0.0001 (5)
C3	0.0181 (6)	0.0189 (5)	0.0209 (6)	0.0020 (5)	0.0013 (4)	0.0018 (5)
C4	0.0185 (6)	0.0166 (5)	0.0226 (6)	0.0008 (4)	0.0018 (4)	−0.0003 (5)
C5	0.0188 (6)	0.0162 (5)	0.0251 (6)	0.0022 (4)	0.0024 (5)	0.0008 (5)
C6	0.0225 (6)	0.0196 (6)	0.0283 (6)	0.0001 (5)	0.0056 (5)	−0.0049 (5)
C7	0.0351 (7)	0.0200 (6)	0.0217 (6)	0.0004 (5)	0.0089 (5)	−0.0059 (5)
C8	0.0473 (9)	0.0292 (7)	0.0242 (6)	−0.0096 (6)	0.0082 (6)	−0.0073 (5)
C9	0.0763 (13)	0.0275 (7)	0.0245 (7)	−0.0166 (8)	0.0113 (8)	−0.0040 (6)
C10	0.0797 (13)	0.0242 (7)	0.0223 (7)	0.0054 (8)	0.0092 (8)	−0.0007 (5)
C11	0.0203 (6)	0.0182 (5)	0.0246 (6)	0.0039 (5)	−0.0010 (5)	0.0023 (5)
C12	0.0342 (7)	0.0259 (6)	0.0263 (6)	0.0032 (6)	0.0067 (5)	−0.0017 (5)
C13	0.0522 (10)	0.0351 (8)	0.0244 (7)	0.0117 (7)	0.0047 (7)	−0.0030 (6)
C14	0.0238 (6)	0.0227 (6)	0.0240 (6)	0.0009 (5)	−0.0033 (5)	−0.0035 (5)

### Geometric parameters (Å, º)

**Table:**

O1—C1	1.4138 (15)	C5—C6	1.5371 (16)
O1—H1A	0.86 (2)	C5—H5	1.0000
O2—C11	1.2333 (16)	C6—C7	1.5138 (18)
O3—C4	1.4210 (14)	C6—H6A	0.9900
O3—H3A	0.93 (2)	C6—H6B	0.9900
N1—C11	1.3279 (17)	C7—C12	1.394 (2)
N1—C14	1.4571 (16)	C7—C8	1.395 (2)
N1—C3	1.4661 (16)	C8—C9	1.392 (2)
C1—C2	1.5390 (18)	C8—H8	0.9500
C1—C14	1.5417 (18)	C9—C10	1.376 (3)
C1—H1	1.0000	C9—H9	0.9500
C2—C3	1.5306 (16)	C10—C13	1.393 (3)
C2—H2A	0.9900	C10—H10	0.9500
C2—H2B	0.9900	C12—C13	1.392 (2)
C3—C4	1.5403 (16)	C12—H12	0.9500
C3—H3	1.0000	C13—H13	0.9500
C4—C5	1.5328 (17)	C14—H14A	0.9900
C4—H4	1.0000	C14—H14B	0.9900
C5—C11	1.5258 (17)		
			
C1—O1—H1A	109.6 (14)	C6—C5—H5	107.3
C4—O3—H3A	107.9 (14)	C7—C6—C5	115.04 (10)
C11—N1—C14	129.85 (11)	C7—C6—H6A	108.5
C11—N1—C3	114.90 (10)	C5—C6—H6A	108.5
C14—N1—C3	114.06 (10)	C7—C6—H6B	108.5
O1—C1—C2	116.78 (11)	C5—C6—H6B	108.5
O1—C1—C14	113.45 (11)	H6A—C6—H6B	107.5
C2—C1—C14	104.54 (10)	C12—C7—C8	118.72 (13)
O1—C1—H1	107.2	C12—C7—C6	120.64 (12)
C2—C1—H1	107.2	C8—C7—C6	120.64 (14)
C14—C1—H1	107.2	C9—C8—C7	120.42 (16)
C3—C2—C1	101.05 (10)	C9—C8—H8	119.8
C3—C2—H2A	111.6	C7—C8—H8	119.8
C1—C2—H2A	111.6	C10—C9—C8	120.42 (15)
C3—C2—H2B	111.6	C10—C9—H9	119.8
C1—C2—H2B	111.6	C8—C9—H9	119.8
H2A—C2—H2B	109.4	C9—C10—C13	119.97 (15)
N1—C3—C2	101.36 (10)	C9—C10—H10	120.0
N1—C3—C4	101.33 (9)	C13—C10—H10	120.0
C2—C3—C4	123.23 (10)	O2—C11—N1	125.35 (12)
N1—C3—H3	109.9	O2—C11—C5	127.58 (11)
C2—C3—H3	109.9	N1—C11—C5	107.07 (11)
C4—C3—H3	109.9	C13—C12—C7	120.81 (14)
O3—C4—C5	110.27 (9)	C13—C12—H12	119.6
O3—C4—C3	114.03 (10)	C7—C12—H12	119.6
C5—C4—C3	102.59 (10)	C12—C13—C10	119.65 (16)
O3—C4—H4	109.9	C12—C13—H13	120.2
C5—C4—H4	109.9	C10—C13—H13	120.2
C3—C4—H4	109.9	N1—C14—C1	101.53 (10)
C11—C5—C4	102.40 (9)	N1—C14—H14A	111.5
C11—C5—C6	114.43 (10)	C1—C14—H14A	111.5
C4—C5—C6	117.51 (10)	N1—C14—H14B	111.5
C11—C5—H5	107.3	C1—C14—H14B	111.5
C4—C5—H5	107.3	H14A—C14—H14B	109.3
			
C(11)—N(1)—C(3)—C(2)	145.23 (10)	H(1)—C(1)—C(2)—C(3)	73
C(11)—N(1)—C(3)—C(4)	17.61 (13)	H(1)—C(1)—C(2)—H(2A)	−169
C(14)—N(1)—C(3)—C(2)	−23.51 (13)	H(1)—C(1)—C(2)—H(2B)	−46
C(14)—N(1)—C(3)—C(4)	−151.12 (10)	O(1)—C(1)—C(14)—H(14A)	−86
C(3)—N(1)—C(11)—O(2)	−176.49 (12)	O(1)—C(1)—C(14)—H(14B)	36
C(3)—N(1)—C(11)—C(5)	3.73 (13)	C(2)—C(1)—C(14)—H(14A)	145
C(14)—N(1)—C(11)—O(2)	−9.9 (2)	C(2)—C(1)—C(14)—H(14B)	−92
C(14)—N(1)—C(11)—C(5)	170.29 (11)	H(1)—C(1)—C(14)—N(1)	−87
C(3)—N(1)—C(14)—C(1)	−1.76 (13)	H(1)—C(1)—C(14)—H(14A)	32
C(11)—N(1)—C(14)—C(1)	−168.41 (12)	H(1)—C(1)—C(14)—H(14B)	154
O(1)—C(1)—C(2)—C(3)	−167.02 (10)	C(1)—C(2)—C(3)—H(3)	−78
C(14)—C(1)—C(2)—C(3)	−40.78 (12)	H(2A)—C(2)—C(3)—N(1)	−81
O(1)—C(1)—C(14)—N(1)	154.80 (10)	H(2A)—C(2)—C(3)—C(4)	31
C(2)—C(1)—C(14)—N(1)	26.51 (12)	H(2A)—C(2)—C(3)—H(3)	163
C(1)—C(2)—C(3)—N(1)	38.05 (11)	H(2B)—C(2)—C(3)—N(1)	157
C(1)—C(2)—C(3)—C(4)	149.83 (11)	H(2B)—C(2)—C(3)—C(4)	−91
N(1)—C(3)—C(4)—O(3)	−149.89 (9)	H(2B)—C(2)—C(3)—H(3)	41
N(1)—C(3)—C(4)—C(5)	−30.67 (11)	N(1)—C(3)—C(4)—H(4)	86
C(2)—C(3)—C(4)—O(3)	98.31 (13)	C(2)—C(3)—C(4)—H(4)	−26
C(2)—C(3)—C(4)—C(5)	−142.47 (11)	H(3)—C(3)—C(4)—O(3)	−34
O(3)—C(4)—C(5)—C(6)	−78.74 (13)	H(3)—C(3)—C(4)—C(5)	86
O(3)—C(4)—C(5)—C(11)	154.95 (10)	H(3)—C(3)—C(4)—H(4)	−158
C(3)—C(4)—C(5)—C(6)	159.44 (10)	O(3)—C(4)—C(5)—H(5)	42
C(3)—C(4)—C(5)—C(11)	33.13 (12)	C(3)—C(4)—C(5)—H(5)	−80
C(4)—C(5)—C(6)—C(7)	−47.18 (15)	H(4)—C(4)—C(5)—C(6)	43
C(11)—C(5)—C(6)—C(7)	72.99 (14)	H(4)—C(4)—C(5)—C(11)	−84
C(4)—C(5)—C(11)—O(2)	156.61 (12)	H(4)—C(4)—C(5)—H(5)	163
C(4)—C(5)—C(11)—N(1)	−23.61 (12)	C(4)—C(5)—C(6)—H(6A)	75
C(6)—C(5)—C(11)—O(2)	28.33 (18)	C(4)—C(5)—C(6)—H(6B)	−169
C(6)—C(5)—C(11)—N(1)	−151.89 (10)	C(11)—C(5)—C(6)—H(6A)	−165
C(5)—C(6)—C(7)—C(8)	−87.85 (15)	C(11)—C(5)—C(6)—H(6B)	−49
C(5)—C(6)—C(7)—C(12)	92.02 (14)	H(5)—C(5)—C(6)—C(7)	−168
C(6)—C(7)—C(8)—C(9)	179.96 (13)	H(5)—C(5)—C(6)—H(6A)	−46
C(12)—C(7)—C(8)—C(9)	0.1 (2)	H(5)—C(5)—C(6)—H(6B)	70
C(6)—C(7)—C(12)—C(13)	−179.84 (12)	H(5)—C(5)—C(11)—O(2)	−91
C(8)—C(7)—C(12)—C(13)	0.0 (2)	H(5)—C(5)—C(11)—N(1)	89
C(7)—C(8)—C(9)—C(10)	−0.4 (2)	H(6A)—C(6)—C(7)—C(8)	150
C(8)—C(9)—C(10)—C(13)	0.6 (2)	H(6A)—C(6)—C(7)—C(12)	−30
C(9)—C(10)—C(13)—C(12)	−0.5 (2)	H(6B)—C(6)—C(7)—C(8)	34
C(7)—C(12)—C(13)—C(10)	0.2 (2)	H(6B)—C(6)—C(7)—C(12)	−146
H(1A)—O(1)—C(1)—C(2)	56.8 (14)	C(6)—C(7)—C(8)—H(8)	0
H(1A)—O(1)—C(1)—C(14)	−64.9 (14)	C(12)—C(7)—C(8)—H(8)	−180
H(1A)—O(1)—C(1)—H(1)	177	C(6)—C(7)—C(12)—H(12)	0
H(3A)—O(3)—C(4)—C(3)	−76.3 (13)	C(8)—C(7)—C(12)—H(12)	−180
H(3A)—O(3)—C(4)—C(5)	168.9 (13)	C(7)—C(8)—C(9)—H(9)	180
H(3A)—O(3)—C(4)—H(4)	48	H(8)—C(8)—C(9)—C(10)	180
C(11)—N(1)—C(3)—H(3)	−99	H(8)—C(8)—C(9)—H(9)	0
C(14)—N(1)—C(3)—H(3)	93	C(8)—C(9)—C(10)—H(10)	−179
C(3)—N(1)—C(14)—H(14A)	−121	H(9)—C(9)—C(10)—C(13)	−179
C(3)—N(1)—C(14)—H(14B)	117	H(9)—C(9)—C(10)—H(10)	1
C(11)—N(1)—C(14)—H(14A)	73	C(9)—C(10)—C(13)—H(13)	179
C(11)—N(1)—C(14)—H(14B)	−50	H(10)—C(10)—C(13)—C(12)	180
O(1)—C(1)—C(2)—H(2A)	−48	H(10)—C(10)—C(13)—H(13)	0
O(1)—C(1)—C(2)—H(2B)	74	C(7)—C(12)—C(13)—H(13)	−180
C(14)—C(1)—C(2)—H(2A)	78	H(12)—C(12)—C(13)—C(10)	−180
C(14)—C(1)—C(2)—H(2B)	−159	H(12)—C(12)—C(13)—H(13)	0

### Hydrogen-bond geometry (Å, º)

**Table:**

*D*—H···*A*	*D*—H	H···*A*	*D*···*A*	*D*—H···*A*
O3—H3*A*···O2^i^	0.93 (2)	1.73 (2)	2.6395 (12)	164 (2)
O1—H1*A*···O3^ii^	0.86 (2)	1.93 (2)	2.7716 (13)	167 (19)

Symmetry codes: (i) *x*+1, *y*, *z*; (ii) −*x*+1, *y*−1/2, −*z*+1/2.
